# Association between peripheral thyroid sensitivity defined by the FT3/FT4 ratio and composite adverse outcome among inpatients with heart failure

**DOI:** 10.3389/fendo.2025.1652749

**Published:** 2025-09-16

**Authors:** Li Ma, Manting Gou, Xingbang Liu, Li Ding, Chao Ma

**Affiliations:** ^1^ Department of Endocrinology and Metabolism, Tianjin Medical University General Hospital, Tianjin, China; ^2^ Department of General Medicine, Heze Hospital Affiliated to Shandong First Medical University, Heze, Shandong, China; ^3^ Department of Endocrinology and Metabolism, Tianjin University Central Hospital, Tianjin, China; ^4^ Department of Cardiology, Heze Municipal Hospital, Heze, Shandong, China; ^5^ Department of Urology, Heze Hospital Affiliated to Shandong First Medical University, Heze, Shandong, China

**Keywords:** FT3/FT4 ratio, heart failure, mortality, readmission, peripheral thyroid sensitivity

## Abstract

**Objective:**

The free triiodothyronine to free thyroxine (FT3/FT4) ratio is an indicator of peripheral thyroid hormone sensitivity. However, its prognostic value in heart failure (HF) remains unclear.

**Methods:**

This single center prospective cohort study included a total of 402 HF patients. The primary composite outcome was established as either mortality from any cause or HF-related hospitalization within one year. Multivariate Cox regression and Kaplan-Meier analysis assessed associations between the FT3/FT4 ratio and composite endpoint risks, with restricted cubic splines (RCS) exploring potential non-linear relationships.

**Results:**

Among 402 heart failure patients, 188 (46.8%) experienced the primary composite endpoint. The highest FT3/FT4 tertile (T3) had 38% lower risk than the lowest tertile (T1) (adjusted HR 0.62, 95% CI 0.41-0.94). In the subgroup of patients with subclinical hypothyroidism (SCH), T3 individuals showed an 84% lower risk compared to T1 (adjusted HR 0.16, 95% CI 0.03–0.81). Both the overall cohort and SCH subgroup exhibited an inverse association between FT3/FT4 ratios and adverse outcomes, whereas euthyroid patients demonstrated a U-shaped relationship with composite endpoint hazards (P for nonlinear = 0.004).

**Conclusions:**

Our findings suggest that maintaining or restoring higher FT3/FT4 levels may improve clinical outcomes in HF patients. Regular monitoring of this ratio, coupled with tailored interventions based on thyroid functional status, could enhance risk stratification and therapeutic decision-making.

## Introduction

Thyroid hormones directly modulate cardiovascular function by enhancing myocardial contractility while also exerting significant indirect effects through sympathetic nervous system activation (1). Even subtle disturbances in thyroid homeostasis are linked to adverse cardiovascular outcomes (2). Heart failure (HF) patients frequently exhibit alterations in thyroid hormone metabolism, including low T3 syndrome and subclinical hypothyroidism (SCH), both established predictors of poor prognosis (3–5). Furthermore, thyrotropin (TSH) levels above 7.0 mU/L show dose-dependent associations with increased coronary heart disease mortality (6, 7), overt hyperthyroidism elevates cardiovascular mortality (8), and high-normal free thyroxine (FT4) correlates with increased mortality in the elderly (9).

Thyroid hormone homeostasis is regulated by the hypothalamic-pituitary-thyroid (HPT) axis (10). Triiodothyronine (T3), the biologically active hormone, is predominantly generated peripherally via deiodination of thyroxine (T4) (11). The FT3/FT4 ratio quantifies peripheral T4-to-T3 conversion efficiency and tissue-level thyroid hormone bioavailability (12, 13), serving as a surrogate marker of peripheral thyroid sensitivity (14, 15). This ratio may be more sensitive than isolated FT3 or FT4 measurements in detecting subtle thyroid metabolic perturbations (16). Emerging evidence supports the FT3/FT4 ratio’s prognostic value in cardiovascular disease for predicting adverse events across multiple populations, including euthyroid acute coronary syndrome patients (17, 18), general cardiovascular disease cohorts (19), and specific groups such as dilated cardiomyopathy (20).

Despite this, the prognostic significance of FT3/FT4 across the spectrum of thyroid function in HF remains underexplored. Notably, HF has an average 1-year mortality rate of up to 33% (21), underscoring the prognostic usefulness for precise, thyroid status-specific risk stratification tools. Defining thyroid function-dependent FT3/FT4 thresholds could enable personalized risk assessment and guide targeted therapies (22).

To address these gaps, this study aimed to: (1) Elucidate the relationship between the FT3/FT4 ratio and composite endpoint risk, and (2) Characterize the nature (linear *vs*. non-linear) and magnitude of associations of the FT3/FT4 ratio with adverse outcomes.

## Methods

### Study population

This prospective cohort study consecutively enrolled 1,300 patients admitted to the Cardiology Department of Heze Hospital, Affiliated to Shandong First Medical University, between January 2022 and December 2023. Inclusion criteria required participants to be ≥18 years old, fulfilling both the 2018 Chinese HF diagnostic guidelines (23) and the 2021 European Society of Cardiology (ESC) HF criteria (24), and New York Heart Association (NYHA) functional class I-IV. Exclusion criteria comprised: (1) unavailable baseline thyroid function data; (2) pre-existing thyroid disorders, malignancy, or severe infections; (3) pregnancy; (4) loss to follow-up; (5) use of thyroid-affecting medications. After exclusions, 402 patients constituted the final analytical cohort. Approved by the Ethics Committee of Heze Hospital Affiliated to Shandong First Medical University (No. 2024-KY001-079). All participants provided written informed consent.

### Data collection

Detailed clinical information was retrieved from the electronic medical records. Clinical information included sex, age, body mass index (BMI), lifestyle factors (smoking, alcohol use), parameters including heart rate (HR), systolic/diastolic blood pressure (SBP/DBP), NYHA class, HF etiology (hypertension, diabetes, coronary heart disease), along with medication records of angiotensin-converting enzyme inhibitors/angiotensin receptor blockers (ACEIs/ARBs), β-blockers, and statins. Venous blood collected within 24 hours of admission underwent analysis for: lipid profiles (total cholesterol [TC], triglycerides [TG], high-density lipoprotein [HDL-C], low-density lipoprotein [LDL-C]), fasting blood glucose [FBG], uric acid [UA], creatinine [Cr], N-terminal pro-B-type natriuretic peptide [NT-proBNP], aspartate aminotransferase [AST], and thyroid function (TSH, FT3, FT4 via direct chemiluminescence; reference ranges: FT3 1.8-4.2 pg/ml, FT4 0.87-1.85 ng/dL, TSH 0.35-5.1 μIU/ml) (Roche Diagnostics Gmbh, Japan). The study population included euthyroid patients and those with thyroid dysfunction (hyperthyroidism, hypothyroidism, subclinical thyroid dysfunction, and other thyroid abnormalities). Euthyroid status is defined as having TSH, FT4, and FT3 all within their respective normal ranges. SCH is characterized by elevated TSH levels (>5.1 uIU/ml) in conjunction with normal FT4. Echocardiography assessed left ventricular ejection fraction (LVEF) and left ventricular end-diastolic dimension (LVDD) using the Biplane Simpson method. All missing values for these covariates are less than 1%.

### Outcomes

The key composite outcome was defined as death from any cause or rehospitalization due to HF within 1 year, selected based on established prognostic relevance in prior studies (25, 26). Endpoint assessors were not blinded to thyroid data.

### Statistical analysis

Statistical analyses were performed using SPSS 25.0 and R 0.5.6 (rms package). Patients were stratified into tertiles based on FT3/FT4 ratio. This equidistant grouping method produced consistent intervals and automatically assigned samples into groups of nearly equal size, due to the continuous distribution of values in the population. Similar approaches have been used in previous studies, such as those by Okoye et al. and Qin et al., which also reported balanced sample distributions across groups as a result of this method (27, 28). Continuous variables were summarized as median and interquartile range (IQR) and compared using the non-parametric Kruskal-Wallis test; categorical variables were presented as counts and percentages, with group comparisons performed using χ² or Fisher’s exact test as appropriate (29). Cox regression was used to assess the association between FT3/FT4 and composite outcomes. Model 1 remained unadjusted, whereas Model 2 incorporated adjustments for sex, age, and BMI. Model 3 added smoking, drinking, SBP, DBP, HR, NYHA class, LVEF, LVDD, TC, TG, LDL-C, HDL-C, FBG, UA, Cr, logNT-proBNP, AST, comorbidities (hypertension, diabetes, coronary heart disease), and HF medications (ACEIs/ARBs, β-blockers, statins). The results are presented as hazard ratios (HRs) along with 95% confidence intervals (CIs). Survival differences were tested with Kaplan-Meier/log-rank methods, while RCS (knots: 10th/50th/90th percentiles) examined nonlinear associations using Model 3 adjustments. Statistical significance threshold was P<0.05.

## Results

### FT3/FT4 ratio in the overall cohort

**Figure 1:**
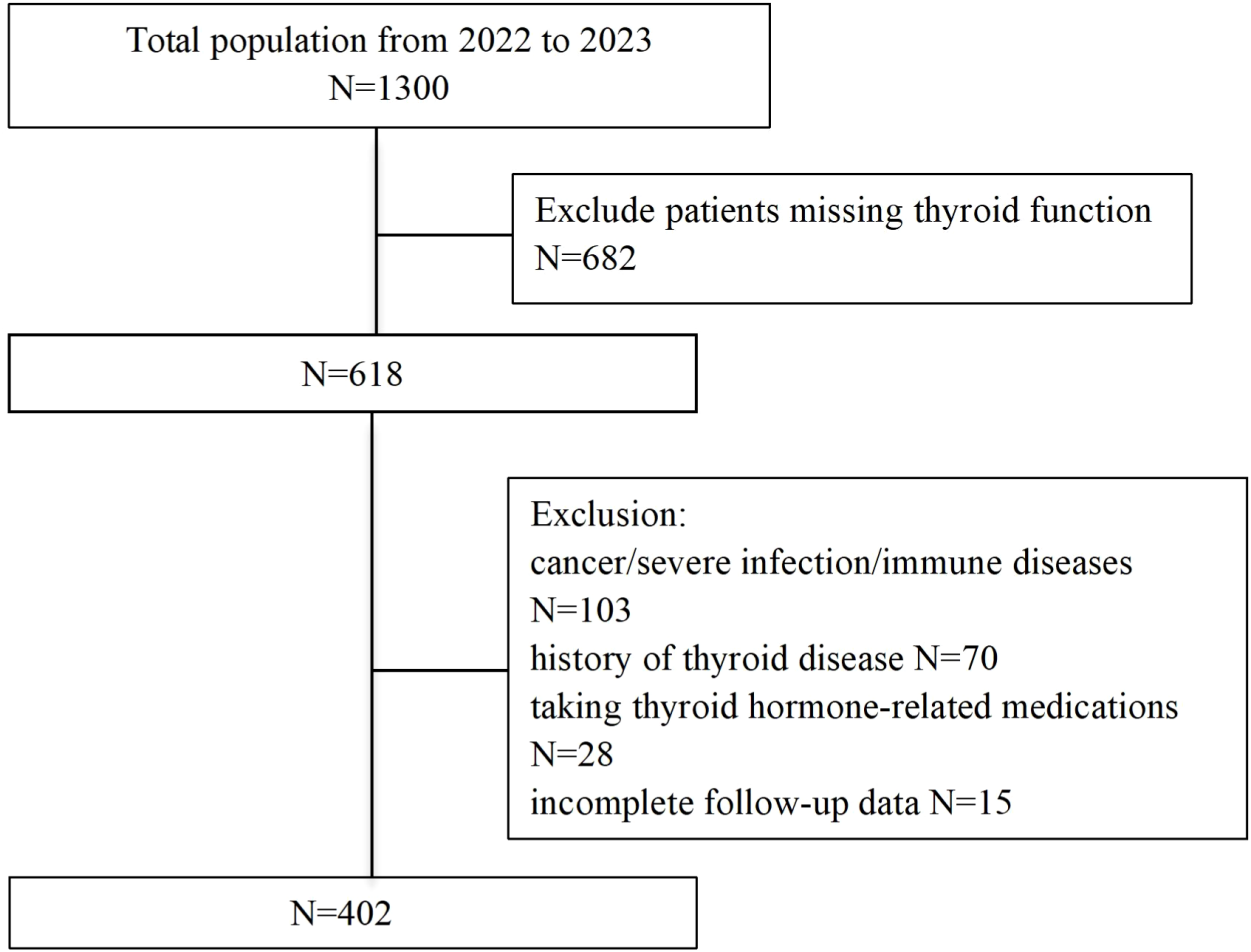
Flow chart of patient selection.

The patient selection flowchart is shown in **Figure 1**. The final analysis included 402 HF patients with a median age of 73 years (IQR: 67-79), of whom 58.0% were male. During follow-up, 188 (46.8%) experienced the composite events. Participants were stratified into tertiles: T1 (≤0.18), (0.18<T2<0.22), and T3 (≥0.22). Baseline characteristics across tertiles are detailed in **Table 1**. Higher FT3/FT4 ratios were associated with elevated BMI, SBP, DBP, LDL-C, and HDL-C levels, alongside lower uric acid, creatinine, NT-proBNP, and AST levels (all P <0.05) (**Table 1**). Notably, the T3 group demonstrated the lowest incidence of composite events (T1: 56.7% vs. T3: 38.1%; P=0.009).

**Table 1:** Baseline characteristics by tertiles of the FT3/FT4 ratio in the main sample (N=402).

FT3/FT4 tertiles	T1 ≤ 0.18 (N=134)	0.18<T2<0.22 (N=134)	T3 ≥0.22 (N=134)	P
Age (years), (IQR)	73 (68, 81)	74 (68, 79)	72 (60, 78)	0.059
Gender, n (%)				
Male	70 (52.2)	76 (56.7)	87 (64.9)	0.103
Female	64 (47.8)	58 (43.3)	47 (35.1)	
BMI (kg/m^2^)	22.6 (20.4, 25.2)	23.4 (20.6, 26.3)	24.0 (21.2, 26.6)	**0.032**
Smoking (%)	30 (22.3)	34 (25.4)	44 (32.8)	0.139
Drinking (%)	16 (11.9)	14 (10.4)	23 (17.2)	0.233
Heart rate (bpm)	86 (70, 100)	89 (73, 103)	84 (72, 98)	0.155
NYHA(III-IV) (%)	119 (88.8)	124 (92.6)	115 (85.8)	0.211
LVEF,%	42 (34, 52)	44 (34, 53)	42 (34, 52)	0.600
LVDD (cm)	56 (48, 62)	56 (49, 64)	57 (50, 64)	0.489
NT-proBNP (ng/L)	9569 (4750, 18942)	5957 (2773, 9144)	3395 (2018, 6820)	**<0.001**
SBP (mmHg)	127 (108, 144)	133 (112, 148)	134 (120, 153)	**0.018**
DBP (mmHg)	78 (68, 89)	83 (75, 91)	81 (73, 91)	**0.044**
FT4 (ng/dl)	1.61 (1.45, 1.84)	1.50 (1.39, 1.63)	1.32 (1.16, 1.48)	**<0.001**
FT3 (pg/ml)	1.84 (1.60, 2.22)	2.44 (2.34, 2.73)	2.85 (2.58, 3.18)	**<0.001**
TSH (uIU/ml)	2.77 (1.74, 4.38)	3.18 (1.92, 5.20)	2.95 (1.87, 4.77)	0.353
TC (mmol/L)	3.47 (2.76, 4.14)	3.53 (3.02, 4.21)	3.83 (3.15, 4.46)	**0.004**
TG (mmol/L)	0.91 (0.72, 1.22)	0.90 (0.71, 1.17)	0.98 (0.72, 1.40)	0.197
LDL-C (mmol/L)	1.93 (1.50, 2.59)	2.15 (1.56, 2.69)	2.17 (1.68, 2.93)	**0.015**
HDL-C (mmol/L)	0.91 (0.75, 1.14)	0.96 (0.81, 1.25)	1.02 (0.84, 1.28)	**0.014**
FBG (mmol/L)	4.75 (4.21, 6.36)	4.78 (4.17, 5.68)	4.90 (4.40, 5.78)	0.491
SUA (umol/L)	420 (294, 519)	325 (259, 404)	327 (274, 429)	**<0.001**
Creatinine (mg/dl)	93 (75, 113)	83 (68, 101)	77 (64, 100)	**<0.001**
AST (U/L)	26 (18, 41)	21 (15, 28)	22 (16, 32)	**0.002**
Hypertension (%)	65 (48.5)	64 (47.8)	73 (54.5)	0.484
Diabetes (%)	43 (32.1)	34 (25.4)	27 (20.1)	0.082
Coronary heart disease (%)	72 (53.7)	63 (47.0)	74 (55.2)	0.358
Statins (%)	94 (70.1)	106 (79.1)	104 (77.6)	0.188
ACEI/ARB (%)	91 (67.9)	96 (71.6)	95 (70.9)	0.779
β-Blocker (%)	100 (74.6)	103 (76.9)	105 (78.4)	0.768
Death or readmission (%)	76 (56.7)	61 (45.5)	51 (38.1)	**0.009**

BMI, body mass index; SBP, systolic blood pressure; DBP, diastolic blood pressure; NYHA, New York Heart Association (NYHA); LVEF, left ventricular ejection fraction; LVDD, left ventricular end diastolic dimension; NT-proBNP, N-terminal pro-B-type natriuretic peptide; FT4, free thyroxine; FT3, free triiodothyronine; TSH, thyroid-stimulating hormone; TC, total cholesterol; TG, triglyceride; LDL-C, low density lipoprotein cholesterol; HDL-C, high density lipoprotein cholesterol; FBG, fasting blood-glucose; SUA, serum uric acid; AST, aspartate aminotransferase; ACEI, angiotensin converting enzyme inhibitor; ARB, angiotensin receptor blocker. Bold indicates P value < 0.05.

**Figure 2:**
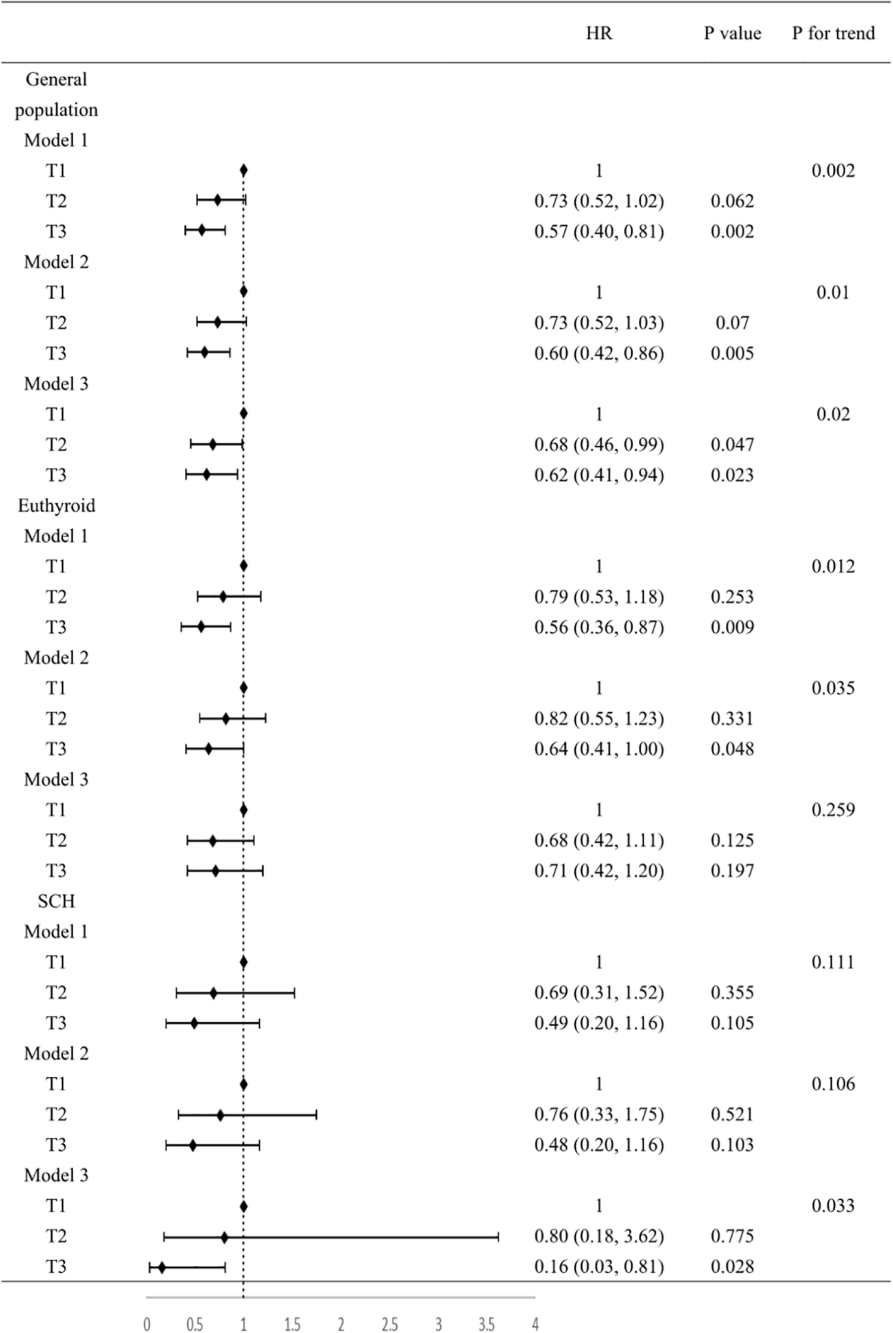
Association between FT3/FT4 value and composite outcomes. Model 1, no covariates were adjusted. Model 2, age, gender, BMI were adjusted. Model 3, age, gender, BMI, smoking, drinking, SBP, DBP, HR, NYHA class, LVEF, LVDD, TC, TG, LDL-C, HDL-C, FBG, UA, Cr, NT-proBNP, AST, ACEI/ARB, β-blockers, statins, hypertension, diabetes, coronary heart disease were adjusted. BMI, body mass index; SBP, systolic blood pressure; DBP, diastolic blood pressure; HR, heart ratio; NYHA, New York Heart Association; LVEF, left ventricular ejection fraction; LVDD, left ventricular end-diastolic dimension; TC, total cholesterol; TG, triglyceride; LDL-C, low-density lipoprotein; HDL-C, high-density lipoprotein; FBG, fasting blood glucose; UA, uric acid; Cr, creatinine; NT-proBNP, N-terminal pro-B-type natriuretic peptide; AST, aspartate aminotransferase; ACEI/ARB, angiotensin-converting enzyme inhibitor/angiotensin receptor blocker.

**Figure 3:**
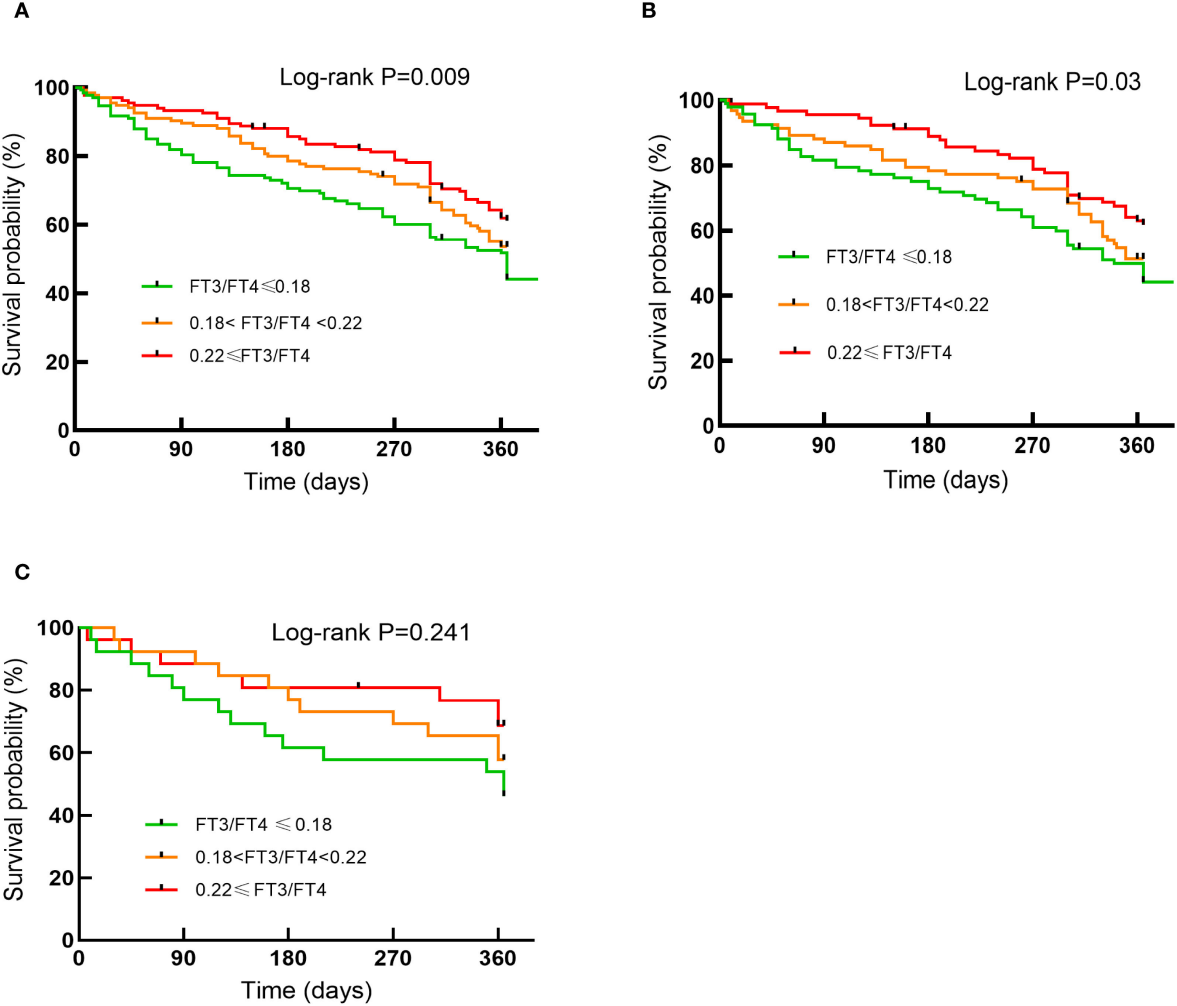
Kaplan-Meier curves for endpoint events by tertiles of FT3/FT4 ratio in the general population **(A)** in the euthyroid population **(B)** in the SCH population **(C)**.

**Figure 4:**
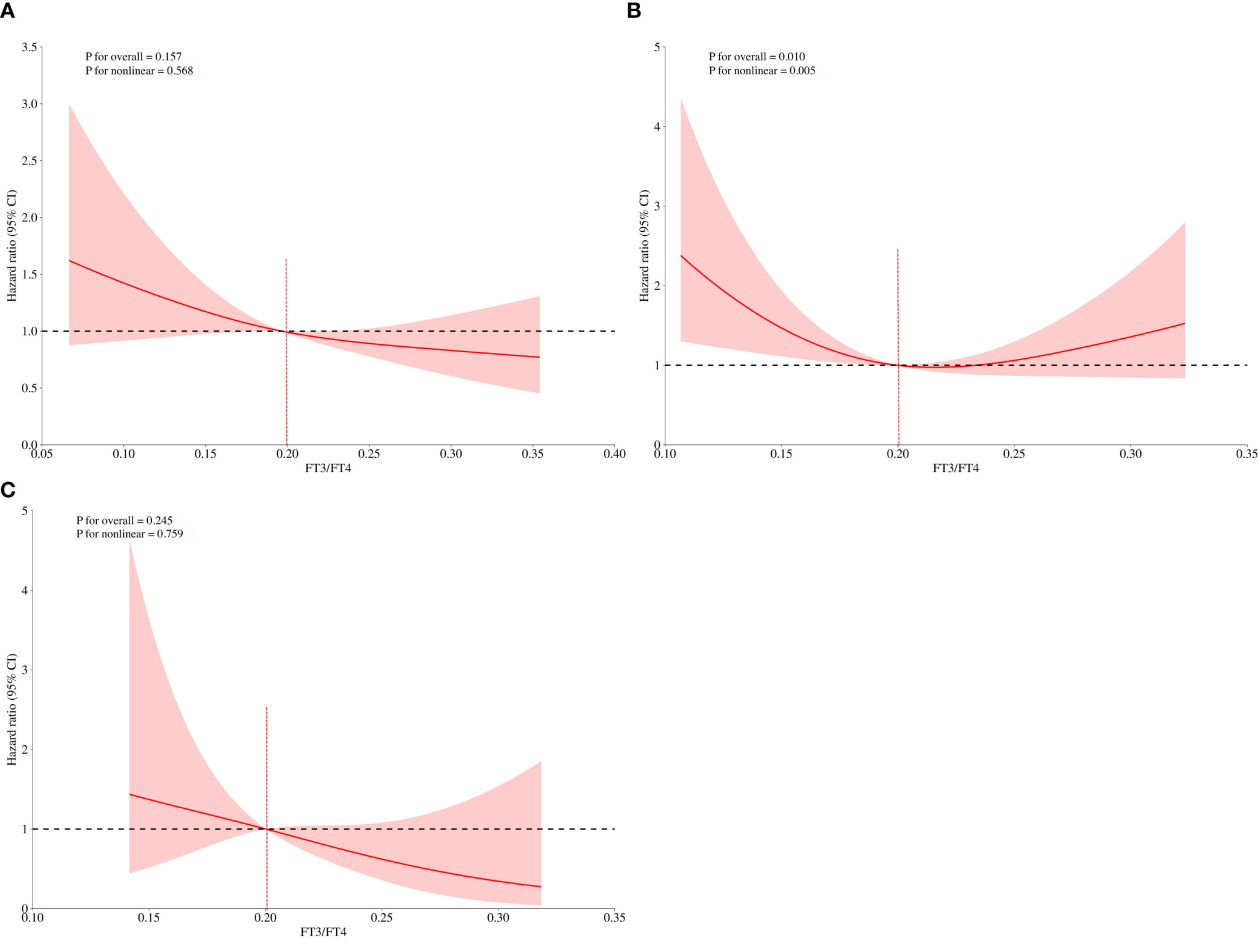
FT3/FT4 and the risk of composite endpoints derived from RCS with 3 knots in the general population **(A)** in the euthyroid population **(B)** in the SCH **(C)**. The dotted lines represent 95% confidence intervals. Spline analyses were adjusted for age, gender, BMI, smoking, drinking, SBP, DBP, HR, NYHA class, LVEF, LVDD, TC, TG, LDL-C, HDL-C, FBG, UA, Cr, NT-proBNP, AST, ACEI/ARB, β-blockers, statins, hypertension, diabetes, coronary heart disease. BMI, body mass index; SBP, systolic blood pressure; DBP, diastolic blood pressure; HR, heart ratio; NYHA, New York Heart Association; LVEF, left ventricular ejection fraction; LVDD, left ventricular end-diastolic dimension; TC, total cholesterol; TG, triglyceride; LDL-C, low-density lipoprotein; HDL-C, high-density lipoprotein; FBG, fasting blood glucose; UA, uric acid; Cr, creatinine; NT-proBNP, N-terminal pro-B-type natriuretic peptide; AST, aspartate aminotransferase; ACEI/ARB, angiotensin-converting enzyme inhibitor/angiotensin receptor blocker.

In Cox regression analyses, both T2 and T3 groups exhibited progressively lower risks of composite outcomes compared to T1. In unadjusted Cox analysis, the T3 group had significantly lower composite risk compared to T1 (HR=0.57, 95% CI 0.40-0.81; P for trend=0.002). Adjustment for sex, age, and BMI (Model 2) yielded similar results. In the fully adjusted model (Model 3), the T2 (HR=0.68, 95%CI 0.46-0.99, P=0.047) and T3 (HR=0.62, 95%CI 0.41-0.94, P=0.023) groups both showed significantly lower risks than the T1 group, with a significant decreasing trend across tertiles (P for trend=0.02) (**Figure 2**). When analyzed as a continuous variable, the FT3/FT4 ratio demonstrated an inverse association with composite outcomes after multivariable adjustment (adjusted HR:0.11, 95%CI: 0.07-0.99, P=0.049). Kaplan-Meier analysis confirmed significantly better event-free survival with higher FT3/FT4 ratios (log-rank P=0.009; **Figure 3A**). RCS analysis demonstrated an inverse association between the continuous FT3/FT4 ratio and composite risk (P for nonlinear=0.568; **Figure 4A**).

### FT3/FT4 ratio in euthyroid population

Of all the participants included in the analysis, 275 had normal thyroid function and a subgroup analysis was performed for this euthyroid cohort. Subjects had a median age of 73 years (IQR: 65-79), with 58.20% being male. A total of 130 composite outcomes were recorded. Baseline characteristics across tertiles (T1 ≤ 0.18; (0.18<T2<0.22); T3≥0.22) are shown in **Table 2**. Elevated FT3/FT4 ratio demonstrated positive associations with BMI, TC, and LDL levels, while correlating inversely with UA, Cr, NT-proBNP, AST, and incidence of composite events (**Table 2**). In addition, euthyroid individuals exhibiting elevated FT3/FT4 ratios were typically younger, predominantly male, and demonstrated higher alcohol consumption prevalence, and elevated LVDD (all P <0.05). Model 1 showed the T3 group exhibited a significant risk reduction in contrast to the T1 group (HR: 0.56, 95% CI 0.36-0.87, P=0.009, P for trend=0.012). This association persisted after sex/age/BMI adjustment in Model 2 (HR: 0.64, 95% CI 0.41-1.00, P=0.048). However, after fully adjusting for variables, no significant association was observed (HR: 0.71, 95% CI 0.42-1.20, P=0.197; **Figure 2**). When analyzed as a continuous variable, the FT3/FT4 ratio showed no significant association with composite outcomes in the multivariable-adjusted model (adjusted HR: 0.17, 95% CI: 0.71–9.72, P=0.394). Kaplan-Meier analysis revealed significant survival disparities across groups (log-rank test: P=0.03, **Figure 3B**). RCS analysis, however, uncovered a U-shaped relationship (P for nonlinear=0.005; **Figure 4B**). The minimum risk was observed when the FT3/FT4 ratio was 0.22 (HR: 0.95, 95% CI: 0.77-1.18).

**Table 2:** Baseline characteristics by tertiles of the FT3/FT4 ratio in the euthyroid samples.

FT3/FT4 tertiles	T1 ≤ 0.18 (N=92)	0.18<T2<0.22 (N=92)	T3≥0.22 (N=91)	P
Age (years), (IQR)	76 (69, 83)	74 (67, 79)	69(60.77)	**<0.001**
Gender, n(%)				
Male	44 (47.8)	52 (56.5)	64 (70.3)	**0.008**
Female	48 (52.2)	40 (43.5)	27 (29.7)	
BMI (kg/m^2^)	22.3 (20.6, 25.2)	23.4 (20.8, 26.3)	24.5 (21.5, 26.7)	**0.029**
Smoking (%)	18 (19.6)	23 (25)	31 (34.1)	0.587
Drinking (%)	9 (9.8)	8 (8.7)	18 (19.8)	**0.046**
Heart rate (bpm)	89 (74, 104)	88 (73, 102)	85 (74, 98)	0.492
NYHA(III-IV) (%)	82 (89.1)	86 (93.5)	77 (84.6)	0.157
LVEF,%	43 (35, 55)	42 (33, 51)	40 (33, 52)	0.351
LVDD (cm)	56 (48, 61)	58 (50, 66)	59 (52, 66)	**0.033**
NT-proBNP (ng/L)	8795 (4461, 19401)	5368 (2563, 8671)	3260 (1952, 6013)	**<0.001**
SBP (mmHg)	127 (108, 142)	136 (108, 152)	130 (120, 147)	0.126
DBP (mmHg)	79 (68, 89)	84 (70, 93)	81 (73, 90)	0.272
FT4 (ng/dl)	1.56 (1.41, 1.67)	1.49 (1.37, 1.62)	1.32 (1.21, 1.48)	**<0.001**
FT3 (pg/ml)	1.92 (1.60, 2.12)	2.45 (2.27, 2.76)	2.88 (2.53, 3.28)	**<0.001**
TSH (uIU/ml)	2.36 (1.48, 3.69)	2.49 (1.64, 3.70)	2.33 (1.61, 3.29)	0.676
TC (mmol/L)	3.38 (2.78, 3.93)	3.72 (3.08, 4.28)	3.68 (3.06, 4.42)	**0.018**
TG (mmol/L)	0.89 (0.72, 1.18)	0.94 (0.70, 1.17)	0.97 (0.72, 1.39)	0.464
LDL-C (mmol/L)	1.92 (1.51, 2.51)	2.19 (1.65, 2.79)	2.15 (1.65, 2.95)	**0.047**
HDL-C (mmol/L)	0.91 (0.77, 1.12)	0.96 (0.79, 1.25)	1.01 (0.79, 1.27)	0.083
FBG (mmol/L)	4.67 (4.26, 6.46)	4.79 (4.31, 5.75)	4.98 (4.42, 5.50)	0.794
SUA (umol/L)	411 (274, 508)	327 (263, 422)	324 (272, 427)	**0.033**
Creatinine (mg/dl)	88 (73, 113)	83 (66, 105)	75 (64, 95)	**0.005**
AST (U/L)	26 (18, 42)	21 (15, 27)	21 (16, 31)	**0.001**
Hypertension (%)	43 (46.7)	45 (48.9)	47 (51.6)	0.801
Diabetes (%)	29 (31.5)	25 (27.2)	16 (17.6)	0.086
Coronary heart disease (%)	47 (51.1)	45 (48.9)	46 (50.5)	0.954
Statins (%)	65 (70.7)	77 (83.7)	72 (79.1)	0.097
ACEI/ARB (%)	64 (69.6)	68 (73.9)	67 (73.6)	0.762
β-Blocker (%)	71 (77.2)	71 (77.2)	74 (81.3)	0.733
Death or readmission (%)	52 (56.5)	44 (47.8)	34 (37.4)	**0.034**

BMI, body mass index; SBP, systolic blood pressure; DBP, diastolic blood pressure; NYHA, New York Heart Association (NYHA); LVEF, left ventricular ejection fraction; LVDD, left ventricular end diastolic dimension; NT-proBNP, N-terminal pro-B-type natriuretic peptide; FT4, free thyroxine; FT3, free triiodothyronine; TSH, thyroid-stimulating hormone; TC, total cholesterol; TG, triglyceride; LDL-C, low density lipoprotein cholesterol; HDL-C, high density lipoprotein cholesterol; FBG, fasting blood-glucose; SUA, serum uric acid; AST, aspartate aminotransferase; ACEI, angiotensin converting enzyme inhibitor; ARB, angiotensin receptor blocker. Bold indicates P value < 0.05.

### FT3/FT4 ratio in SCH population

The SCH subgroup included 78 patients with median age 75 years (IQR:68-80); 57.8% male), with 33 composite events (42.3%). Increasing FT3/FT4 ratios predicted increased SBP and NT-proBNP levels (P<0.05) (**Table 3**). In Model 3, the T3 group (≥0.22) had a substantially lower composite risk compared to T1 (≤0.18) (HR: 0.16, 95% CI 0.03–0.81; P=0.028) with progressive risk attenuation (P for trend=0.033; **Figure 2**). When analyzed as a continuous variable, the FT3/FT4 ratio demonstrated an inverse association with composite outcomes after multivariable adjustment (adjusted HR:0.45, 95%CI: 0.23-0.87, P=0.018). Kaplan-Meier survival curves showed no significant difference (log-rank P=0.241; **Figure 3C**), but RCS analysis demonstrated an inverse correlation (P for nonlinear=0.759; **Figure 4C**).

**Table 3:** Baseline characteristics by tertiles of the FT3/FT4 ratio in the SCH samples.

FT3/FT4 tertiles	T1 ≤ 0.18 (N=26)	0.18<T2<0.22 (N=26)	T3≥0.22 (N=26)	P
Age (years), (IQR)	74(70,80)	72(67,79)	77(72,83)	0.324
Gender, n (%)				
Male	12 (46.2)	18 (69.2)	15 (57.7)	0.242
Female	14 (53.8)	8 (30.8)	11 (42.3)	
BMI (kg/m^2^)	22.5 (20.3, 25.6)	22.9 (19.9, 24.4)	23.7 (19.5, 26.3)	0.675
Smoking (%)	5 (19.2)	7 (26.9)	8 (30.8)	0.625
Drinking (%)	2 (7.7)	4 (15.4)	5 (19.2)	0.477
Heart rate (bpm)	84 (71, 92)	90 (78, 108)	84 (70, 103)	0.524
NYHA(III-IV) (%)	24 (92.3)	23 (88.5)	24 (92.3)	0.855
LVEF, %	46 (36, 58)	44 (37, 54)	46 (40, 55)	0.845
LVDD (cm)	56 (47, 60)	56 (51, 63)	52 (48, 59)	0.492
NT-proBNP (ng/L)	9898 (5624, 16957)	6036 (2580, 9655)	5671 (2076, 8868)	**0.005**
SBP (mmHg)	126 (109, 147)	128 (114, 140)	151 (119, 172)	**0.017**
DBP (mmHg)	78 (70, 90)	81 (76, 87)	81 (70, 97)	0.605
FT4 (ng/dl)	1.54 (1.45, 1.73)	1.51 (1.28, 1.56)	1.22 (1.09, 1.34)	**<0.001**
FT3 (pg/ml)	2.09 (1.90, 2.27)	2.46 (2.20, 2.63)	2.82 (2.58, 2.99)	**<0.001**
TSH (uIU/ml)	6.31 (5.47, 8.53)	7.26 (6.12, 10.39)	7.66 (6.45, 12.67)	0.087
TC (mmol/L)	3.68 (2.57, 4.55)	3.30 (2.85, 4.14)	4.01 (3.13, 4.47)	0.264
TG (mmol/L)	0.92 (0.64, 1.32)	0.89 (0.77, 1.21)	0.94 (0.71, 1.39)	0.869
LDL-C (mmol/L)	1.73 (1.32, 2.92)	1.74 (1.45, 2.32)	2.21 (1.80, 2.74)	0.143
HDL-C (mmol/L)	1.03 (0.68, 1.26)	0.97 (0.86, 1.31)	1.10 (0.96, 1.39)	0.252
FBG (mmol/L)	4.67 (4.00, 5.97)	4.62 (3.93, 5.17)	5.16 (4.32, 7.13)	0.270
SUA (umol/L)	381 (294, 478)	320 (262, 385)	386 (273, 484)	0.299
Creatinine (mg/dl)	96 (82, 114)	83 (70, 101)	94 (67, 150)	0.091
AST (U/L)	26 (18, 37)	19 (15, 26)	22 (15, 31)	0.092
Hypertension (%)	16 (61.5)	10 (38.5)	17 (65.4)	0.108
Diabetes (%)	10 (38.5)	7 (26.9)	7 (26.9)	0.582
CHD (%)	14 (53.9)	12 (46.2)	16 (61.5)	0.538
Statins (%)	21 (80.8)	17 (65.4)	20 (76.9)	0.417
ACEI/ARB (%)	15 (57.7)	19 (73.1)	17 (65.4)	0.507
β-Blocker (%)	20 (76.9)	19 (73.1)	18 (69.2)	0.822
Death or readmission (%)	14 (53.8)	11 (42.3)	8 (30.8)	0.110

BMI, body mass index; SBP, systolic blood pressure; DBP, diastolic blood pressure; NYHA, New York Heart Association (NYHA); LVEF, left ventricular ejection fraction; LVDD, left ventricular end diastolic dimension; NT-proBNP, N-terminal pro-B-type natriuretic peptide; FT4, free thyroxine; FT3, free triiodothyronine; TSH, thyroid-stimulating hormone; TC, total cholesterol; TG, triglyceride; LDL-C, low density lipoprotein cholesterol; HDL-C, high density lipoprotein cholesterol; FBG, fasting blood-glucose; SUA, serum uric acid; AST, aspartate aminotransferase; CHD, coronary heart disease ACEI, angiotensin converting enzyme inhibitor; ARB, angiotensin receptor blocker. Bold indicates P value < 0.05.

### FT3/FT4 ratio in the remaining 49 patients

Among the remaining 49 patients, the median age was 74 (IQR:65-78) years, 57.1% were male, and 25 composite endpoint events were recorded. Baseline characteristics across tertiles are detailed in **Supplementary Table 1**. Since our initial exclusion criteria applied only to patients with pre-existing thyroid disease, rather than those with newly identified dysfunction in this study, the 49 patients were included in the overall population but were not analyzed separately due to limited sample size, so as not to compromise statistical power in subgroup analyses.

## Discussion

This prospective study demonstrates that the FT3/FT4 ratio, a marker of peripheral thyroid hormone sensitivity, exhibits thyroid status-dependent associations with 1-year mortality rehospitalization risk in heart failure patients. Our key novel findings are: (1) An inverse relationship was observed between the FT3/FT4 ratio and composite risk across the overall HF cohort, particularly among patients with SCH; (2) A significant U-shaped relationship is observed in euthyroid HF patients.

HF is characterized by metabolic derangements including reduced nutrient intake (30), chronic inflammation, and oxidative stress (31), which can impair micronutrient absorption (iodine, selenium, zinc, iron) crucial for thyroid hormone synthesis and conversion (32). Thyroid hormones, in turn, profoundly impact cardiac electrophysiology, contractility, and structure (33). Hence, Subtle fluctuations in thyroid hormone bioavailability can therefore significantly influence cardiac function and HF progression (34). The inverse FT3/FT4 ratio-adverse outcome relationship likely involves multifactorial pathways. Reduced ratios indicate impaired peripheral T4-to-T3 conversion, directly contributing to tissue-level hypothyroidism during acute/chronic disease states (18). T3, acting via nuclear receptors (TRα/TRβ) in the heart, enhances the expression of key proteins involved in calcium handling (SERCA2a, RYR2) and mitochondrial function/biogenesis; processes often compromised in HF (35). Lower FT3 levels directly contributes to HF pathogenesis by impairing left ventricular relaxation and increasing myocardial stiffness (36). Furthermore, HF is associated with sympathetic overactivation and Renin-Angiotensin-Aldosterone System (RASS) hyperactivity (37); T3 restores autonomic balance in heart failure by: (1) Attenuating sympathetic overactivation through downregulation of myocardial β adrenergic receptor density and reduction of circulating norepinephrine levels (38); (2) Recovering baroreflex function via upregulation of neuronal nitric oxide synthase (nNOS) in the nucleus tractus solitarius (39), thereby mitigating these detrimental neurohormonal axes (40).

Our findings align with and extend previous research. Studies linked lower FT3/FT4 ratios to increased mortality in dilated cardiomyopathy (20). A study using propensity matching demonstrated that a reduced ratio of FT3/FT4 is a robust predictor of all-cause mortality in heart failure patients (41). Another study discovered that the FT3/FT4 ratio independently predicts all-cause mortality in the general population. In addition, a study noted that in euthyroid patients with type 2 diabetes mellitus, a low FT3/FT4 ratio independently contributed to major adverse cardiac events following acute myocardial infarction (42). However, our study is the first to comprehensively evaluate this ratio across distinct thyroid functional states (euthyroid vs. SCH) within an HF cohort and to identify state-specific risk patterns. The protective association observed in SCH patients is particularly noteworthy and suggests that maintaining adequate peripheral conversion is crucial in this subgroup, potentially outweighing the risks associated with mildly elevated TSH. This finding resonates with experimental data showing T3 reduces infarct size and activates cardio-protection during ischemia/reperfusion (43), and clinical evidence that low-dose T3 replacement improved LV function post-AMI (44). Notably, in patients with SCH, multivariate Cox regression demonstrated an inverse association between the FT3/FT4 ratio and the risk of composite outcomes, whereas KM analysis revealed no significant survival difference among the three groups. This discordance likely reflects the inherent limitation of KM analysis: as a non-parametric method estimating unadjusted survival probabilities from observed events, it does not account for confounding factors.

The U-shaped relationship observed in euthyroid patients is a novel and significant finding. The loss of significance in the fully adjusted multivariate Cox model for the euthyroid subgroup, particularly after accounting for age and NT-proBNP-both strongly associated with the ratio and outcomes, suggesting that the univariate association was partly confounded. This interpretation is primarily informed by the simultaneous measurement of NT-proBNP and thyroid indicators during the same blood draw, which inherently precludes establishing the temporal sequence required for mediation (where exposure must precede the mediator, which subsequently influences the outcome) (45). However, the highly significant U-curve revealed by RCS analysis indicates a complex, non-monotonic relationship. This suggests an optimal range for peripheral thyroid sensitivity in euthyroid HF. Ratios significantly below 0.22 likely indicate impaired conversion and tissue hypothyroidism, increasing risk. Conversely, ratios significantly above this point might reflect excessive peripheral conversion, potentially linked to hypermetabolic states or impaired hormone clearance, which could paradoxically increase cardiovascular strain or indicate other underlying metabolic disturbances detrimental in HF. Compensatory mechanisms preserving tissue-level thyroid hormone action within the normal functional range might also buffer the impact of ratio variations, except at the extremes (46). Potential mechanisms involve: (1) impaired enzymatic efficiency due to the Thr92Ala DIO2 polymorphism at low FT4/FT3 ratios, whereas DIO2 overexpression under high-T4 conditions depletes essential cofactors (e.g., glutathione), thereby exacerbating T4-to-T3 conversion failure (47); (2) pro-inflammatory cytokines (TNF-α, IL-6) suppressing deiodinase activity through NF-κB-mediated downregulation of DIO1 and DIO2 expression, impairing T3 generation and promoting thyroid hormone resistance during chronic inflammation (48). Further research is needed to elucidate the mechanisms underlying this U-shape.

To our knowledge, this is the first study to identify the optimal FT3/FT4 for stratifying mortality/readmission risk in HF patients with varying thyroid states. Limitations should be acknowledged. First, the single-center design necessitates cautious interpretation regarding generalizability. As iodine sufficiency varies significantly across global regions, and Asian populations often exhibit distinct iodine nutritional status compared to Western cohorts, our results should be interpreted within this contextual framework. Second, only baseline thyroid function was assessed; serial measurements might better capture dynamic changes relevant to prognosis. Third, the mechanisms linking the FT3/FT4 ratio to HF outcomes, particularly the U-shape in euthyroidism, require further elucidation. Fourth, In the SCH cohort, the observed hazard ratio (HR) of 0.16 should be interpreted with caution due to the limited sample size. Finally, observational design precludes causal inference.

In conclusion, the FT3/FT4 ratio is a thyroid status-dependent predictor of 1-year mortality and HF rehospitalization risk. Monitoring the FT3/FT4 ratio, offers a valuable tool for risk stratification. Future studies should validate these thresholds in diverse populations and explore whether interventions aimed at optimizing peripheral thyroid hormone sensitivity.

## Data Availability

The original contributions presented in the study are included in the article/**Supplementary Material**. Further inquiries can be directed to the corresponding authors.

## Supplementary material

Supplementary Table 1 Baseline characteristics of the remaining 49 patients by FT3/FT4 tertiles.
