# Supplementary material for: Association between peripheral thyroid sensitivity defined by the FT3/FT4 ratio and composite adverse outcome among inpatients with heart failure
Source: Front Endocrinol (Lausanne). 2025 Sep 16;16:1652749. doi: 10.3389/fendo.2025.1652749 (PMC12479335; doi:10.3389/fendo.2025.1652749)
Supplement: Supplementary Table 1 — Baseline characteristics of the remaining 49 patients by FT3/FT4 tertiles. [file Table1.docx]

**Supplementary Table 1.** Baseline characteristics of the remaining 49 patients by FT3/FT4 tertiles

BMI: body mass index; SBP: systolic blood pressure; DBP: diastolic blood pressure; NYHA: New York Heart Association (NYHA); LVEF: left ventricular ejection fraction; LVDD: left ventricular end diastolic dimension; NT-proBNP: N-terminal pro-B-type natriuretic peptide; FT4: free thyroxine; FT3: free triiodothyronine; TSH: thyroid-stimulating hormone; TC: total cholesterol; TG: triglyceride; LDL-C: low density lipoprotein cholesterol; HDL-C: high density lipoprotein cholesterol; FBG: fasting blood-glucose; SUA: serum uric acid; AST: aspartate aminotransferase; CHD: coronary heart diseaseACEI: angiotensin converting enzyme inhibitor; ARB: angiotensin receptor blocker.

| FT3/FT4 tertiles | T1≤0.14  (N=17) | 0.14<T2<0.19 (N=16) | T3≥0.19  (N=16) | P |
| --- | --- | --- | --- | --- |
| Age (years), (IQR) | 71 (57, 76) | 73 (65,79) | 76 (69, 81) | 0.195 |
| Gender, n (%) |  |  |  |  |
| Male | 9 (52.9) | 12 (75) | 7 (43.8) | 0.185 |
| Female | 8 (47.1) | 4 (25) | 9 (56.3) |  |
| BMI (kg/m^2^) | 23.5 (19.1, 27.6) | 23.0 (20.2, 24.7) | 23.4 (21.1, 25.4) | 0.832 |
| Smoking (%) | 4 (23.5) | 6 (37.5) | 6 (37.5) | 0.625 |
| Drinking (%) | 3 (17.6) | 2 (12.5) | 1 (6.3) | 0.477 |
| Heart rate (bpm) | 72 (61, 98) | 92 (74, 125) | 85 (71, 96) | 0.095 |
| NYHA(III-IV) (%) | 13 (76.4) | 15 (93.8) | 13 (81.3) | 0.386 |
| LVEF, % | 36 (26, 44) | 39 (30, 52) | 46 (40, 55) | **0.038** |
| LVDD (cm) | 57 (51, 67) | 55 (50, 66) | 51 (44, 57) | 0.133 |
| NT-proBNP (ng/L) | 10134 (4721, 28877) | 7304 (3110, 16044) | 4414 (2251, 7998) | **0.045** |
| SBP (mmHg) | 116 (107, 146) | 134 (114, 154) | 140 (122, 154) | 0.275 |
| DBP (mmHg) | 78 (67, 90) | 81 (63, 100) | 82 (75, 97) | 0.566 |
| FT4 (ng/dl) | 2.22 (2.06, 2.41) | 1.98 (1.92, 2.05) | 1.46 (0.74, 2.22) | **0.003** |
| FT3 (pg/ml) | 1.75 (1.02, 2.41) | 2.81 (2.47, 2.92) | 2.81 (2.17, 3.99) | **<0.001** |
| TSH (uIU/ml) | 2.42 (1.68, 4.40) | 3.30 (1.92, 5.77) | 4.76 (0.04, 30.91) | 0.671 |
| TC (mmol/L) | 3.51 (2.89, 4.03) | 3.56 (2.88, 4.35) | 3.92 (3.18, 5.08) | 0.444 |
| TG (mmol/L) | 0.99 (0.83, 1.26) | 0.82 (0.70, 1.17) | 0.88 (0.61, 1.62) | 0.275 |
| LDL-C (mmol/L) | 2.22 (1.51, 2.68) | 2.24 (1.59, 2.54) | 2.43 (1.47, 3.25) | 0.809 |
| HDL-C (mmol/L) | 0.89 (0.75, 1.10) | 0.94 (0.89, 1.30) | 0.99 (0.85, 1.20) | 0.341 |
| FBG (mmol/L) | 5.01 (4.39, 6.22) | 5.00 (4.15, 5.48) | 4.65 (4.03, 5.88) | 0.653 |
| SUA (umol/L) | 450 (340, 638) | 428 (376, 590) | 332 (250, 423) | **0.040** |
| Creatinine (mg/dl) | 96 (74, 123) | 98 (78, 107) | 74 (57, 96) | **0.039** |
| AST (U/L) | 25 (19, 37) | 25 (22, 36) | 26 (18, 42) | 0.938 |
| Hypertension (%) | 6 (35.3) | 8 (50) | 10 (62.5) | 0.294 |
| Diabetes (%) | 4 (23.5) | 3 (18.8) | 3 (18.8) | 0.925 |
| CHD (%) | 10 (58.8) | 7 (43.8) | 11 (68.8) | 0.355 |
| Statins (%) | 11 (64.7) | 11 (68.8) | 10 (62.5) | 0.931 |
| ACEI/ARB (%) | 12 (70.6) | 12 (75) | 8 (50) | 0.283 |
| β-Blocker (%) | 10 (58.8) | 13 (81.2) | 12 (75) | 0.336 |
| Death or readmission (%) | 8 (47.1) | 9 (56.3) | 8 (50) | 0.866 |
